# Between single ion magnets and macromolecules: a polymer/transition metal-based semi-solid solution†
†Electronic supplementary information (ESI) available: structural data for the Co-based SIM compounds; photographs of the obtained mononuclear crystalline compounds; comparison of microelemental analysis results and ICP + MS for **1**, **2**, example bulk P4VP with CoBr_2_, and P4VP alone; Fourier-transform infrared spectroscopy (FTIR) measurement results for **1**, **2**, bulk P4VP with CoBr_2_ and P4VP alone; P4VP with CoBr_2_ geometry optimization results; AC magnetic susceptibility *vs.* frequency for **1** and **2**; AC magnetization *versus* frequency for the bulk CoBr_2_–P4VP; thin film roughness from AFM measurements; XPS analysis for unmodified and modified P4VP thin films and the bulk P4VP with CoBr_2_; ellipsometry measured film thicknesses; SIMS supplementary data for the obtained films; crystallographic information file with the structures of the mononuclear compounds **1** and **2** (cif). CCDC 1811487 and 1811488. For ESI and crystallographic data in CIF or other electronic format see DOI: 10.1039/c8sc02277a


**DOI:** 10.1039/c8sc02277a

**Published:** 2018-08-03

**Authors:** Anna M. Majcher, Paweł Dąbczyński, Mateusz M. Marzec, Magdalena Ceglarska, Jakub Rysz, Andrzej Bernasik, Shin-ichi Ohkoshi, Olaf Stefańczyk

**Affiliations:** a Faculty of Physics, Astronomy and Applied Computer Science , Jagiellonian University , Łojasiewicza 11 , 30-348 Krakow , Poland . Email: anna.majcher@uj.edu.pl; b Academic Centre for Materials and Nanotechnology , AGH University of Science and Technology , al. Mickiewicza 30 , 30-049 Kraków , Poland; c Faculty of Physics and Applied Computer Science , AGH University of Science and Technology , al. Mickiewicza 30 , 30-049 Kraków , Poland; d Department of Chemistry , School of Science , The University of Tokyo , 7-3-1 Hongo, Bunkyo-ku , Tokyo 113-0033 , Japan, Email: olaf@chem.s.u-tokyo.ac.jp

## Abstract

The creation of functional magnetic materials for application in high-density memory storage or in the new field of molecular spintronics is a matter of widespread interest among the material research community. Herein, we describe a new approach that combines the qualities of single ion magnets, displaying slow magnetic relaxations, and the merits of polymers, being easy to process and widely used to produce thin films. Basing the idea on cobalt(ii) ions and pyridine-based single ion magnets, a new macromolecular magnetic material was obtained – a polymeric matrix of poly(4-vinylpyridine) (P4VP) cross-linked by a cobalt(ii) salt bound within it, effectively forming a network of single ion magnets, with field-induced magnetic relaxations preserved in both bulk and thin film forms. The binding of cobalt is confirmed by a series of methods, like secondary ion mass spectroscopy or high-resolution X-ray photoelectron spectroscopy. The magnetic relaxation times, up to 5 × 10^–6^ s, are controllable simply by dilution, making this new material a semi-solid solution. By this approach, a new path is formed to connect molecular magnetism and polymer science, showing that the easy polymer processing can be used in forming self-organizing functional magnetic thin films.

## Abstract 2

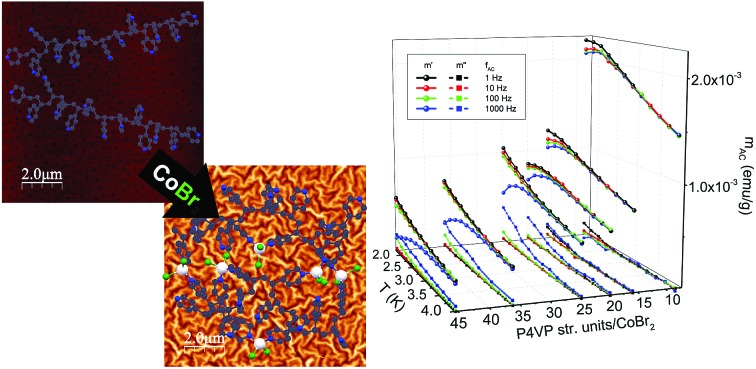
A new material combining polymers and magnetic relaxations both in the bulk solid solution and in the thin film form.

## Introduction

Every technological revolution has its roots in basic research. This thought has long been the dream fuelling the scientists who devote their efforts to synthesizing and studying the properties of low dimensional molecular magnetic materials that display slow relaxations of magnetization:^1^ Single Ion Magnets (SIMs),^2^–^4^ Single Molecule Magnets (SMMs)^5^ and Single Chain Magnets (SCMs).^6^ Such low-dimensional systems can be based on not only the historically first transition metals, like cobalt, but recent focus has been on lanthanide-based SMMs that tend to exhibit higher blocking temperatures and energy barriers.^7^,^8^

Efforts have been made to incorporate such systems into layers (mostly the first SMM Mn_12_Ac and its variations). To do that, two main strategies can be employed: chemisorption and physisorption. Historically, the latter was first – Mn_12_ clusters have been incorporated into Langmuir–Blodgett films, exhibiting a hysteresis of molecular origin.^9^,^10^ Electrospray ion beam deposition of Mn_12_ was done on insulating surfaces.^11^,^12^ The deposition of Mn_12_ complexes was also done from solution by a series of methods (breath-figure or lithographically controlled wetting as well as chemisorption by chemical modification of the complex and/or surface).^13^ The topographical pattern of a DVD was recreated using a solution of polymer and Mn_12_ molecules.^14^ Self-organized growth of Co ferromagnetic clusters of roughly 15 atoms on a Pt surface was done by deposition from a vapour phase.^15^ Other than the one mentioned^13^ the reported examples of using chemisorption to form layers of low-dimensional molecular magnets are: chemical grafting of Fe_4_ SMMs^16^ or Tb-based SMMs^17^ to surfaces. TbPc_2_ SMMs have successfully been coupled to carbon nanotubes by supramolecular interactions.^18^ Surface anchoring of Co(ii) and Ni(ii) complexes has also recently been done.^19^ The recent focus has been not only on memory storage but also, even more importantly, on molecular spintronics.

Our research, presented herein, takes a different approach: we have combined the qualities of macromolecules that are widely used to produce macromolecular thin films, and the characteristics of Single Ion Magnets (SIMs) that exhibit slow magnetic relaxations. This work proves that by incorporating a transition metal-based SIM into a polymeric matrix we can preserve or even enhance the magnetic relaxations crucial for using the SIM as an information carrier, while placing it in a material that can protect and stabilize the metal within and is easy to process. This was done both in the bulk form and in the form of a macromolecular magnetic thin film. Although in this particular case the temperature at which the relaxations happen is low, and the relaxation times are yet far from making the SIMs being able to store bytes of memory; the fact that single ion magnets and macromolecules together form a new class of materials is truly ground-breaking.

There have been numerous efforts to incorporate metals into polymers at the stage of polymerization, which prompted the creation of a new class of materials, called metallopolymers.^20^,^21^ There has also been a report of using block copolymers to form compounds with Prussian blue analogs;^22^ they have, however, never been studied from the point of view of their magnetism, not to mention magnetic relaxations. Another clue that prompted our research was the fact that P4VP-coated carbon nanotubes have been used for magnetic wastewater purification.^23^

Our approach was as follows: after synthesizing mononuclear cobalt(ii) compounds formed with the use of simple ligands, in this case pyridine and 4-vinylpyridine, and confirming that they are single ion magnets, we employed an analogical macromolecule with its structural units identical to the ligands that form the studied SIM – the already polymerized poly(4-vinylpyridine). This is a fundamental difference between the material that we obtained and metallopolymers, as in the latter the metal is incorporated at the stage of polymerization.

## Experimental

### Materials

All reagents were purchased from Sigma Aldrich or Alfa Aesar and used without further purification. Anhydrous cobalt(ii) bromide was handled in an inert atmosphere due to its extreme sensitivity to humidity.

**Caution!** Cobalt(ii) bromide like 4-vinylpyridine is toxic and should be handled with care.

### Syntheses of mononuclear compounds

Both syntheses were inspired by an already long-known synthesis.^24^

#### Co(py)_2_Br_2_ (**1**)

1 mmol of anhydrous CoBr_2_ (218.7 mg) was dissolved in 5 ml of absolute ethanol resulting in a clear blue solution. 2 mmols of pyridine (161 μl) were then added. After about ten minutes, dark blue crystals began to form. The product was collected by filtration and washed with a small quantity of cold absolute ethanol. Average yield: around 80% based on the amount of Co (≈300 mg). The pristine product was used immediately for further experiments to prevent the eventual change of composition. Crystallites of **1** are stable under ambient conditions for a long time; however, the finely powdered assembly quickly absorbs humidity which results in the formation of a pink-coloured powder of Co(py)_2_(H_2_O)_2_Br_2_.^25^

#### Co(4vpy)_2_Br_2_ (**2**)

The synthesis was analogous to the previous case. After adding 2 mmol of 4-vinylpyridine (215 μl) to a solution of 1 mmol CoBr_2_ in 5 ml of ethanol, fine blue crystals formed almost instantly. Average yield: around 73% based on the amount of Co (≈315 mg).

Crystallites of **2** were found to be much more stable in contrast to assembly **1** and even the finely powdered assembly does not change composition for several days in an ambient atmosphere.

### Crystal structure determination

Single crystal X-ray diffraction data for **1** and **2** were collected at room temperature on a Rigaku R-AXIS RAPID diffractometer equipped with an imaging plate area detector using graphite monochromated Mo-K_α_ radiation (*λ* = 0.71075 Å). Single crystals suspended in paratone-N oil were mounted with a 100 μm Dual Thickness Micro Mount™ loop. The intensity data were integrated by using Rigaku RAPID AUTO. Structures were solved by direct methods using SHELXS-97 (ref. 26) incorporated in the CrystalStructure 4.0 crystallographic software package^27^ and refined using a full-matrix least squares technique of SHELXL-2014/7 (ref. 28) included in the OLEX-2 1.2 software package.^29^

Non-hydrogen atoms were refined anisotropically while hydrogen atoms were positioned with an idealized geometry and refined using a riding model. Crystal data, data collection, and refinement parameters for **1** and **2** are listed in Table 1. The structural data presented as figures were prepared with the use of CCDC Mercury 3.9 visualization software.^30^

**Table 1:** Crystal data, data collection, and refinement parameters for **1** and **2**

	**1**	**2**
Molecular formula	C_10_H_10_Br_2_CoN_2_	C_14_H_14_Br_2_CoN_2_
*M*_r_ [g mol^–1^]	376.95	429.02
*T* (K)	296(2)	296(2)
Radical used, *λ* (Å)	Mo Kα (0.71075)	
Crystal system, space group	Monoclinic; *P*2_1_/*c*	Monoclinic; *C*2/*c*
*a* [Å]	8.6842(10)	9.1275(7)
*b* [Å]	18.1757(18)	12.1835(8)
*c* [Å]	8.5209(8)	14.8264(10)
*α* [°]	90	90
*β* [°]	100.878(7)	95.764(7)
*γ* [°]	90	90
*V* [Å^3^]	1320.8(2)	1640.4(2)
*Z*, *d*_calcd_ (g cm^–3^)	4, 1.896	4, 1.737
*μ* (mm^–1^)	7.320	5.906
*F*(000)	724	836
2*θ* range for data collection (°)	5.276–54.912	5.524–54.932
Index ranges	–11 ≤ *h* ≤ 11	–11 ≤ *h* ≤ 11
	–22 ≤ *k* ≤ 23	–15 ≤ *k* ≤ 15
	–9 ≤ *l* ≤ 11	–19 ≤ *l* ≤ 17
Reflections collected/unique	6969/2987 (*R*_int_ = 0.0742, *R*_sigma_ = 0.1003)	12 807/1870 (*R*_int_ = 0.0449, *R*_sigma_ = 0.0273)
Refinement method	Full-matrix least-squares on *F*^2^	
Data/restraints/parameters	2987/0/136	1870/0/105
Goodness-of-fit on *F*^2^	1.250	1.107
Final *R* indices (*I* > 2*σ*(*I*))	*R*_1_ = 0.1064, w*R*_2_ = 0.0813	*R*_1_ = 0.0515, w*R*_2_ = 0.0651
*R* indices (all data)	*R*_1_ = 0.1847, w*R*_2_ = 0.0925	*R*_1_ = 0.1037, w*R*_2_ = 0.0753
Largest diff. peak/hole (e Å^–3^)	0.58/–0.41	0.29/–0.25

Powder X-ray diffraction patterns of the polycrystalline samples of **1** and **2** were collected on a Rigaku Ultima-IV equipped with Cu-K_α_ radiation (*λ* = 1.5418 Å). For these measurements, single crystals of **1** and **2** were ground and packed into the sample holder.

### Syntheses of bulk P4VP with CoBr_2_

Adequate amounts of P4VP and CoBr_2_ were dissolved in anhydrous methanol separately (in concentrations no higher than 50 mg ml^–1^ for the polymer and 5–20 mg ml^–1^ for the salt). Then, under vigorous stirring, the cobalt salt solution was pipetted into the polymer solution. The immediate precipitation of a threadlike blue product was visible. After being left to settle for at least one hour at room temperature, the product was then collected by means of a pipette from the bottom of the vial and dried under lowered pressure for several hours. The resulting blue powder, the colour intensity of which depended on the cobalt concentration, was stable under ambient conditions in the vast majority of cases, regardless of the time left in air.

### Microelemental analysis and spectroscopic methods

Elemental analysis was performed by standard microanalysis on an Elementar Analysensysteme GmbH: vario MICRO cube analyzer and ICP + MS was done by means of an Agilent 7700 inductively coupled plasma mass spectrometer (ICP + MS). Infrared absorption spectra were recorded with a JASCO IRT-3100 FT-IR microscope.

### Polymer and P4VP–CoBr_2_ structure modelling and visualization

Possible molecular arrangements for P4VP and P4VP with CoBr_2_ were obtained by using the 3D optimization algorithm implemented in ACD/ChemSketch software.^31^ Geometry optimization of one P4VP chain coordinating to one CoBr_2_ unit was performed using ArgusLab software.^32^ This was done by fixing the number *n* of structural units between the units coordinating to Co and calculating the minimum potential energy of all implemented atoms and bonds. The Hamiltonian used was the Molecular Mechanics UFF (Universal Force Field) available within the software. Attempts to perform simulations for larger numbers of P4VP chains and/or CoBr_2_ units did not deliver any reliable results (the structures did not converge); however, we believe that the simple model used is sufficient to explain the findings presented within this article. The results of the calculations for *n* = 0 to 6 and structure visualizations can be found in the ESI in Table 4.† Chemical structures were visualized using CCDC Mercury 3.9 software.^30^

### Preparation of thin macromolecular magnetic films

Thin films of P4VP were obtained by spin-casting (2000 rpm) a 20 mg ml^–1^ polymeric solution in chloroform onto cleaned silica wafers. Several resulting layers were then treated with acetonitrile (previously tested not to dissolve the polymer) for 60 seconds to ensure that the final effect is not caused by the orthogonal solvent alone. Several P4VP layers were immersed in a 20 mg per ml CoBr_2_ solution in acetonitrile for 60 seconds and then rinsed with pure acetonitrile. The film was then dried in an inert atmosphere. The set of all films, as-cast, treated with acetonitrile alone and CoBr_2_ solution-treated, was examined using all the available methods except for magnetic measurements, for which the thin polymeric film was produced using a home-built H-dipping^33^ setup on thin plastic foil to minimize the diamagnetic contribution to the signal. The thin film area was approximately 5 times 10 cm^2^, and the film was later treated with CoBr_2_ acetonitrile solution identically to the previous case.

### Magnetic measurements

All magnetic measurements were performed using a Quantum Design MPMS 5XL system, with the available range of temperatures 1.8–400 K and fields up to 50 kOe. All dry bulk products – SIM compounds and macromolecular magnetic materials – were pressed inside gelatine capsules and fitted into the measurement straw without using additional adhesives. The measurement of a thin macromolecular magnetic film was performed by compressing a layer of size 5 cm × 10 cm produced by H-dipping on plastic foil into a size suitable to fit within the plastic straw used for standard measurements. All DC measurements were corrected for the diamagnetic contribution of the sample holders and constituent atoms (Pascal's tables).^34^

The frequency dependence of the AC susceptibility was analysed using the following Cole–Cole model:^35^1
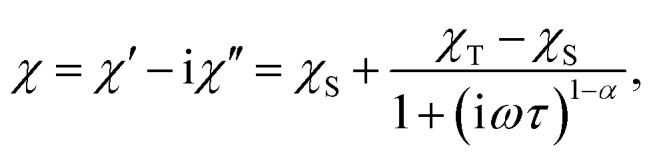
which was fitted to the in-phase and out-of-phase magnetization simultaneously in each case. The resulting values of *τ* were then plotted as their logarithms *versus* inverse temperature, and a weighted linear fit of the Arrhenius law was performed for each case, thereby yielding the values of *τ*_0_ and the heights of the energy barrier for specific concentrations of Co atoms within the macromolecular matrix. All the data obtained from magnetic measurements were processed using OriginPro 9.1 software.

The concentration of Co for each sample was estimated by assuming low-temperature magnetization (1.8 K) in 50 kOe identical to the total magnetic moment value produced by CoBr_2_(py)_2_ under the same conditions. The validity of this method was cross-checked with the concentrations calculated from microelemental analysis and ICP + MS to show perfect agreement (see the ESI† for details).

### Atomic force microscopy

AFM images of film surface topographies were obtained at room temperature with a Nanosurf FlexAFM microscope working in contact mode. Setpoints and gains were adjusted for each measurement to obtain a clear image without noise. Each film topography was investigated by collecting images at several randomly chosen areas. The images were processed using WSxM 4.0 software.^36^

### X-ray photoelectron spectroscopy

The XPS analyses were carried out in a PHI VersaProbeII Scanning XPS system using monochromatic Al Kα (1486.6 eV) X-rays focused on a 100 μm spot and scanned over the sample area of 400 μm × 400 μm. The photoelectron take-off angle was 45° and the pass energy in the analyzer was set to 23.50 eV to obtain high energy resolution spectra for the C 1s, N 1s and Co 2p regions. A dual beam charge compensation with 7 eV Ar^+^ ions and 1 eV electrons was used to maintain a constant sample surface potential regardless of the sample conductivity. All XPS spectra were charge referenced to the unfunctionalized, saturated carbon (C–C) C 1s peak at 284.8 eV. The operating pressure in the analytical chamber was less than 4 × 10^–9^ mbar. The deconvolution of spectra was performed using PHI MultiPak software (v.9.7.0.1). The spectrum background was subtracted using the Shirley method.

### Time-of-flight: secondary ion mass spectrometry

The TOF-SIMS experiments were performed on an ION:TOF TOF:SIMS V (Munster, Germany) instrument, equipped with a liquid metal ion source and caesium ion source. Static SIMS measurements were carried out with the use of a Bi_3_^+^ 30 keV ion beam. Both negative and positive spectra were collected. Depth profiles of samples were obtained in dual beam mode. A 1keV Cs^+^ ion beam was used to sputter a 500 × 500 μm^2^ area and a Bi_3_^+^ 30 keV ion beam was used to analyse a 100 × 100 μm^2^ area concentric to the sputtered surface.

### Ellipsometry

The thin film thicknesses were measured by using a spectroscopic ellipsometer (SENTECH SpectraRey/3) equipped with a micro-spot. The ellipsometric angles *Ψ* and *Δ* were determined for wavelengths *λ* in the spectral range between 320 and 800 nm. The measurements were taken at angles of incidence and detection of 70°. The polymer layers were modelled as a Cauchy layer of thickness *d* with a refractive index *n*(*λ*) of the form:

2 *n*(*λ*)=*n*_0_ + *n*_1_*λ*^–2^ + *n*_2_*λ*^–4^.

Layer thickness and the parameters *n*_0_, *n*_1_ and *n*_2_ were varied numerically in order to achieve the best agreement between the model and the measured values *Δ*(*λ*) and *Ψ*(*λ*). The thickness of the silicon oxide layer on the top of the substrate was measured independently and accounted for in the model.

## Results and discussion

### Syntheses of the mononuclear compounds

**Fig. 1:**
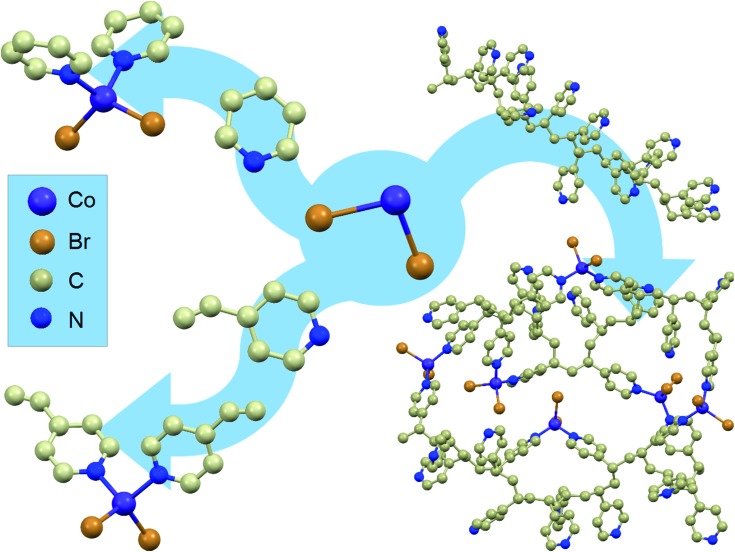
Synthetic routes for obtaining the described materials: combining CoBr_2_ salt (middle) with pyridine leads to mononuclear compound **1** (top left arrow) and with 4-vinylpyridine to mononuclear compound **2** (bottom left arrow). Reaction of poly(4-vinylpyridine) with CoBr_2_ leads to the formation of the new macromolecular magnetic material (right arrow). Structures of **1** and **2** were obtained *via* single crystal XRD measurements while P4VP and P4VP–CoBr_2_ were modelled using the geometry optimization algorithm in ACD/ChemSketch software. Hydrogen atoms are omitted for clarity.

Starting from cobalt(ii) salts, pyridine and 4-vinylpyridine, two mononuclear compounds were synthesised (**1** and **2**). Their synthesis has been known for many years;^37^ however, they have never been investigated for the occurrence of magnetic relaxations despite their tetrahedral geometry.^4^ The scheme of the synthesis and the structure visualizations of the obtained compounds can be found in Fig. 1 on the left. For details of the synthesis and structure visualization, see the Experimental section. The single crystal structures of compounds **1** and **2** consist of mononuclear distorted tetrahedral CoL_2_Br_2_ complexes, where L is pyridine for **1** and 4-vinylpyridine for **2** (a more detailed structural diagram can be found in Fig. 1 in the ESI†). It is worth emphasizing that in both structures there are no crystallization solvent molecules. Furthermore, none of the assemblies show any hydrogen bonds, π–π or anion–π interactions that would form a long-range network, which is uncommon for molecular materials with aromatic organic ligands and which provides good isolation of the single molecules from each other within the crystal.

### Magnetic properties of the mononuclear compounds

Measurements of alternating current (AC) magnetic susceptibility proved the existence of field-induced (*H*_DC_ = 2.5 kOe) slow magnetic relaxations, typical of single ion magnets (Fig. 3, ESI†). The Cole–Cole model was fitted to the real and imaginary components of AC magnetic susceptibility *χ*′ and *χ*′′ simultaneously, delivering the height of the energy barriers equal to 28(2) and 35(3) K, and relaxation time *τ*_0_ values of 8 × 10^–10^ s and 1 × 10^–11^ s for **1** and **2**, respectively. The *α* parameter that describes the distribution of relaxation times ranged from 0.13 to 0.28 and from 0.027 to 0.36 for **1** and **2**, respectively, which is well within the range typical of single ion magnets (for details see the ESI†).

### Syntheses of the macromolecular magnetic material

The same approach was undertaken to prepare a magnetic macromolecular matrix (synthesis visualized in Fig. 1, right). Structural characterization was not possible in this case as there is no ordered crystal structure – powder X-ray diffraction showed a single amorphous peak (not shown). The visualizations of P4VP and P4VP with CoBr_2_ in Fig. 1 were obtained by geometry optimization (for details, see the Experimental section and ESI†). Detailed calculations performed on a simple model of one CoBr_2_ unit coordinating to one P4VP chain in different geometries (varying numbers of structural units between the units coordinating to the Co atom) showed that concentrations higher than *ca*. 4 P4VP structural units in one chain coordinating to one cobalt would be energetically unfavourable. This explains the results of the reactions at a high Co:P4VP structural unit molar ratio, resulting in the maximal achievable effective cobalt concentration no higher than one cobalt per *ca*. 5 P4VP structural units (for details of the performed syntheses, see the Experimental section and ESI†). The solution of cobalt salt added to a solution of poly(4-vinylpiridine) in the same solvent causes an immediately visible precipitation of a blue threadlike product indicating the cross-linking of the polymer. The product of the reaction – bright blue powder – by colour itself suggests the binding of the cobalt ion in tetrahedral geometry,^38^ the same as in the mononuclear compounds. The material, dried under lowered pressure, is stable under ambient conditions, which is another very important feature of this substance, as tetrahedral cobalt complexes are often unstable in air and susceptible to oxygen and moisture. The reaction was performed with varying molar ratios (from 1 mole of cobalt salt per two moles of polymer structural units up to 1 : 40). As the amount of the cross-linked polymer was difficult to control, the cobalt concentration in the final product was in each case estimated by comparing the low-temperature high-field magnetization with the value produced by **1**. The validity of this method of Co amount estimation was checked by microelemental analysis and ICP + MS (for details, see the ESI†).

### High resolution X-ray photoelectron spectroscopy of the bulk

Doping the polymer with CoBr_2_ produces a new, highly energetic nitrogen N 1s peak at a binding energy of about 400 eV (Fig. 5, ESI†) which has previously been detected for P4VP doped with either Pd^39^ or Pt^40^ complexes, and was ascribed to nitrogen atoms coordinated to these metals.

### Magnetic properties of the macromolecular magnetic material

**Fig. 2:**
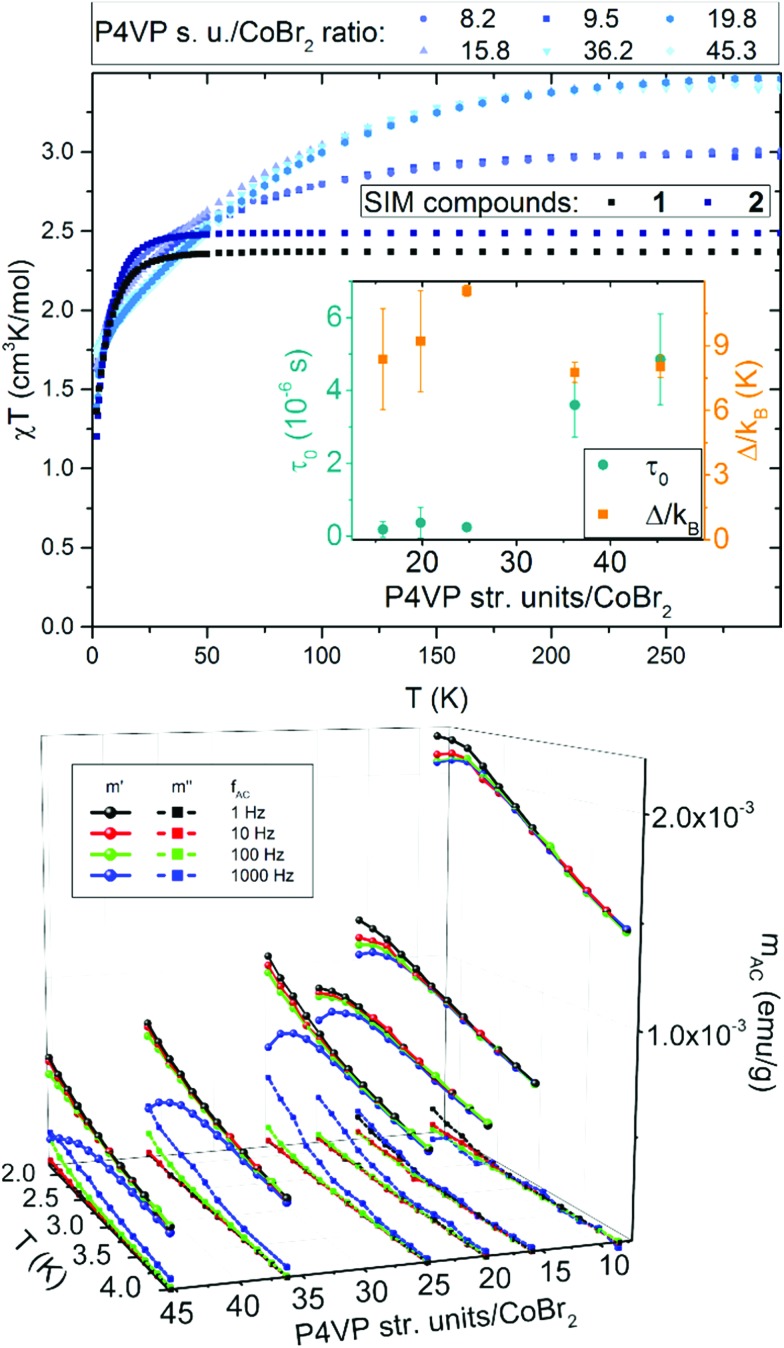
Magnetic properties of the bulk new material: results are presented for bulk P4VP with CoBr_2_ at varying P4VP structural units : Co molar ratios. Top: *χT vs. T* (measured in 1 kOe) additionally compared with the same data for **1** and **2**. Bottom: 3D plot of AC magnetization measured in a DC field of 2.5 kOe at four different frequencies as a function of temperature and of the molar ratio of P4VP structural units per one CoBr_2_. *H*_AC_ = 3 Oe in each case. Top inset: phase diagram of the *τ*_0_ value and height of the energy barrier as a function of the P4VP structural units per one CoBr_2_ molar ratio.

Magnetic measurements for a series of P4VP with CoBr_2_ both in constant and alternating magnetic fields revealed several surprising features. Magnetization measured in static magnetic field of 1 kOe, depicted as *χT* per one cobalt(ii) ion *vs. T* in Fig. 2 on the top show that, compared to the results for **1** and **2** (*χT* at room temperature equal 2.4 and 2.5 cm^3^ K mol^–1^, respectively, within the range typical for similar SIMs). However, for the macromolecular magnetic material *χT* behaves in a different way, gradually reaching higher values at higher temperatures. What is even more interesting is that there is an abrupt change from around 3.0 cm^3^ K mol^–1^ at room temperature for high cobalt concentrations (below 10 P4VP structural units per one CoBr_2_) to almost 4.0 cm^3^ K mol^–1^ for lower concentrations of cobalt (more than 10 P4VP structural units per one CoBr_2_). Both these observations can be explained by the fact that polymer chains become elastic at high temperatures, which allows the anisotropic cobalt ions to orient towards the direction of the applied magnetic field. This hypothesis would also explain the higher *χT* values for lower cobalt concentrations – the fewer the Co atoms that cross-link the polymeric matrix, the less rigid the network is and the more freely the cobalt ions can orient towards the field direction without breaking the chemical bonds in the structure. This is suppressed at low temperatures (below 100 K), where the polymer chains freeze and their elasticity decreases. It should be noted that the decrease in *χT* for all the compounds observed at low temperatures is typical of all Co(ii) compounds and is due to single-ion anisotropy.^41^

AC magnetic measurements revealed other interesting features of this material. Firstly, the field-induced (*H*_DC_ = 2.5 kOe) magnetic relaxations of Co(ii) are indeed preserved within the polymeric matrix, with one slight, but very important improvement – a visible drop in the *α* parameter, which in every case stayed within the range 0.15–0.20. This in average is comparable to or lower that the values for **1** and **2**. This means that despite a completely disordered structure of the polymeric matrix, the distribution of the relaxation times for all Co(ii) centres is very narrow. The second intriguing property of this new material is that with lowering the concentration of the cobalt centres within the polymer, a significant increase of the relaxation times can be observed.

The results of the AC magnetic measurements in a DC field of 2.5 kOe are presented in Fig. 2 (bottom). Each measurement of *m*_AC_*versus* temperature was performed at four different frequencies of the alternating magnetic field. The difference between the values of 
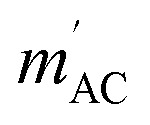
 for different frequencies and the value of 
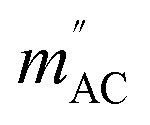
, small for high Co concentrations, becomes notably larger with increasing Co dilution. This means that at low temperatures the lower the concentration of Co centres within the polymer, the slower the magnetic relaxations become. Such features are often seen in the so-called solid solutions.^42^ However, as our material does not have an ordered crystal structure, we will call it a semi-solid solution – as we are on the border of soft matter and ordered molecular compounds. Fits of the Arrhenius law to plots of the natural logarithm of relaxation times (each value being a result of a Cole–Cole model fit to isothermal AC susceptibility frequency dependence – see Fig. 4 in the ESI†) *versus* inverse temperature produced interesting results concerning the height of the energy barrier *Δ*/*k*_B_ and the *τ*_0_ parameter – depicted as the inset in Fig. 2 (top). The pattern that emerges is as follows: decreasing cobalt concentration has a much less significant influence on the height of the energy barrier, which keeps the same order of magnitude and shows no monotonous change, than on the relaxation time – at higher ratios of P4VP structural units per one cobalt ion, and the value of *τ*_0_ increases significantly. A similar effect has already been observed for K(Ph_4_P)[Co_0.06_Zn_0.94_(OPh)_4_], which required dilution with diamagnetic species to induce slow magnetic relaxations.^43^ Here, the mechanism is different in the sense that the polymer chains are flexible – therefore, they can effectively adjust to coordinate the CoBr_2_ units in almost any way, and no diamagnetic salt is needed in order to achieve the desired dilution.

### Preparation of thin macromolecular magnetic films

As the main goal of this research was obtaining a thin macromolecular film that would combine the magnetic properties of SIM compounds with the qualities of polymers (such as ease of processing), the same reaction was reproduced as described herein. Because the product of the previous synthesis is a cross-linked polymer network which is impossible to dissolve without its destruction, a different approach was employed. First, we produced thin films of the polymer alone. This step was followed by immersing the produced layers in a solution of cobalt salt in a solvent orthogonal to the polymer (acetonitrile). The layer was then washed with the same solvent to ensure that no unbound cobalt salt remained in the produced films. To exclude the effect of the solvent, a layer of P4VP treated with acetonitrile only was also produced for comparison.

### Atomic force microscopy (AFM) of the thin films

**Fig. 3:**
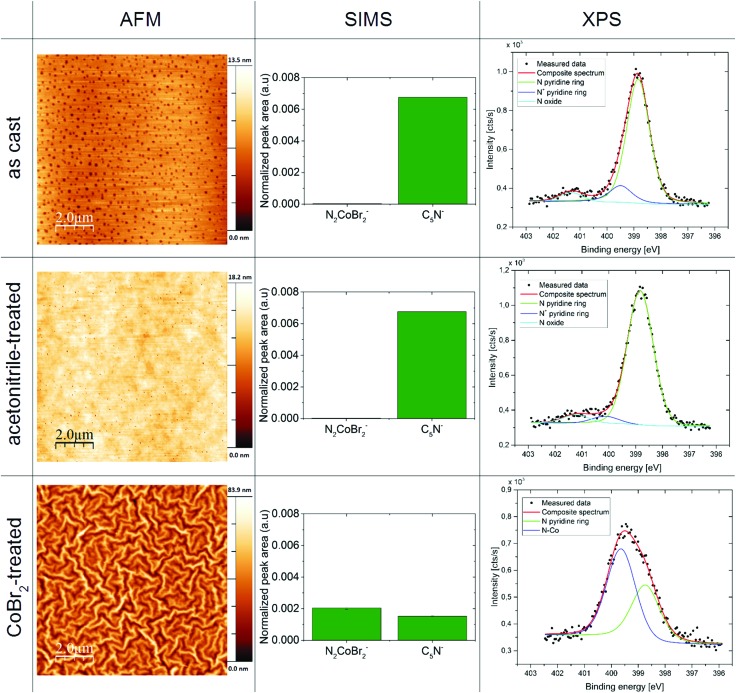
Properties of thin films of the macromolecular magnetic material compared with films of P4VP as-cast and treated with the orthogonal solvent only: representative results of atomic force microscopy measurements (left column), secondary ion mass spectroscopy (middle column) and high resolution X-ray photoelectron spectroscopy N 1s spectra (right column) for thin films of P4VP as-cast (top row), P4VP treated with acetonitrile alone (middle row) and a P4VP film treated with CoBr_2_ acetonitrile solution (bottom row).

AFM was used to characterize the film topography. As can be seen in Fig. 3 in the left column, the films processed with the use of the cobalt salt reveal a tenfold increase in the surface roughness (see Table 5 in the ESI† for roughness analysis results), compared to the P4VP as-cast and treated with acetonitrile only. The pasta-shaped features in the topography of the CoBr_2_-doped P4VP film are consistent with the wrinkling of a cross-linked rigid layer on a layer of a softer polymer.^44^,^45^

### Secondary ion mass spectrometry (SIMS) of the thin films

**Fig. 4:**
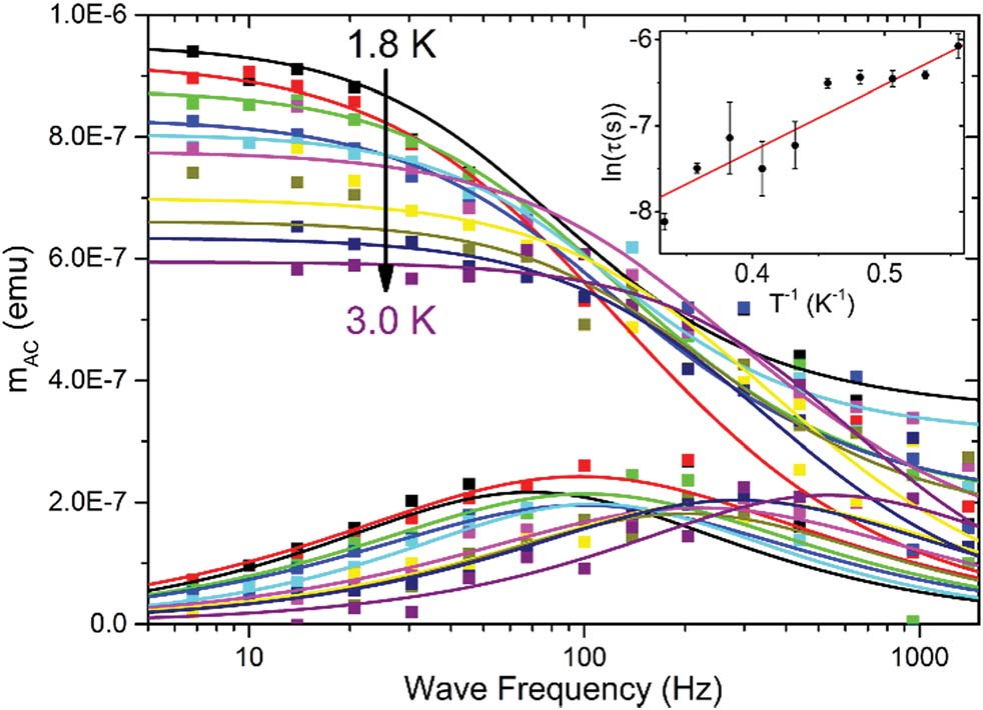
AC magnetization measured in *H*_DC_ = 2.5 kOe *versus* AC field frequency for a thin film of P4VP treated with CoBr_2_ solution. *H*_AC_ = 3 Oe. Solid lines represent respective Cole–Cole model fits performed simultaneously for 
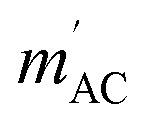
 and 
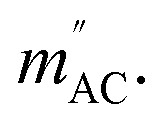
 Inset: relaxation times obtained from the Cole–Cole fits as a function of inverse temperature with a linear Arrhenius law fit.

The SIMS data for the obtained films deliver a confirmation that CoBr_2_ is bound to the pyridine rings within the polymeric layer (see Fig. 4, middle column). There is a notable increase of CoBr_2_N_2_^–^ units in CoBr_2_ solution-treated films compared to the films as-cast and treated with acetonitrile. This suggests the binding of the cobalt centre to the nitrogen atoms found in the pyridine structural units. This is supported by a sharp drop in the number of unbound pyridines (seen as the decrease of C_5_N^–^ units in CoBr_2_ solution-treated films compared to untreated samples). For more results of the SIMS measurements, please see the ESI.† Depth profiling shows that only the surface layer is occupied by cobalt ions (no more than 10% of the total film thickness in this case – thickness was determined by an ellipsometry measurement, for details see the Experimental section and ESI†).

### High-resolution X-ray photoelectron spectroscopy of the thin films

The XPS data provided an insight into the chemical bonds within the obtained magnetic macromolecular films. An example of the obtained high-resolution XPS spectra for nitrogen 1s regions can be seen in Fig. 4 in the right column. A modification of the N 1s band in the sample treated with the cobalt salt solution compared with the samples as-cast and treated with the orthogonal solvent only – which agrees with the data obtained for the bulk product (Fig. 7, ESI†) – is consistent with Co atoms coordinating nitrogen in pyridine units. For a more detailed insight into the XPS results, please see the ESI.†

### Magnetic properties of the macromolecular magnetic thin film

To investigate the magnetic properties, a large-area P4VP film was produced on thin plastic foil and treated with CoBr_2_ solution to ensure the amount of cobalt needed to produce a satisfactory signal. Measurements of AC magnetization delivered a confirmation that the cobalt centres within the film show field-induced (*H*_DC_ = 2.5 kOe) magnetic relaxations (Fig. 4). The relaxation times derived from Cole–Cole model fits plotted logarithmically as a function of inverse temperature (Fig. 4 inset) with a linear fit performed on them deliver a rough estimate of the *τ*_0_ value of 3 × 10^–6^ s and the height of the energy barrier 8(2) K, the order of magnitude of which stays within the pattern that emerged for the bulk samples. The considerable errors are caused by the fact that this measurement was done on the verge of the magnetometer sensitivity (10^–8^ emu compared with the 10^–7^ emu signal); however, the qualitative confirmation that field-induced relaxations are also preserved in the form of a thin film is undeniable. In order to probe the electronic structure of such thin films it would generally be useful to employ X-ray absorption spectroscopy and its derivative methods (X-ray Magneto-Chiral Dichroism and X-ray Natural Linear Dichroism) to obtain information on both orbital and spin components of the magnetic moment – however, the short relaxation times will most probably prevent getting reliable information with these methods.^46^ Using our data, it is also impossible to obtain information on the orientation of magnetic centres within the layer; however, it is highly unlikely that the produced films are epitaxial. Obtaining films with a more oriented magnetic structure is another challenge which we will attempt to overcome in near future – the research presented herein is just a starting point.

## Conclusions

Taking into account all the findings presented within this article, we conclude that we discovered a new way to obtain functional materials that combine the qualities of single ion magnets (field-induced magnetic relaxations) and polymers (with the emphasis on the ease of processing). We also show an easy way to form thin films of such a material without the loss of properties. By a series of methods, we proved that CoBr_2_ binds to the pyridine units in the polymer, both in the bulk and in the form of a thin film. A thin film of polymer, physisorbed to an Si surface, chemisorbs the cobalt salt within – thus joining the two strategies of forming thin films of low-dimensional molecular magnets. The magnetic relaxations of the Co centres, controllable by simple dilution, are preserved and even enhanced in this new material. What is also not to be underestimated is the stability of such a material – the polymer acts as a protective agent to the single ion magnet that becomes a part of a macromolecular matrix. However, perhaps the most important finding is that the synthesis of the described macromolecular magnetic material in both forms is very simple. Therefore, this result is a large step towards the application of single ion magnets as, *e.g.*, bytes of computer memory, which has long been the goal of scientists working with these materials.

We believe that our findings open a new path that combines two major fields of material research – polymer science and molecular magnetism – which, until now, had few common elements. Such a connection is extremely promising regarding future applications of molecular magnetic materials – as self-organizing macromolecular magnetic materials, which are a new class by themselves.

## Conflicts of interest

The authors declare no competing financial interests.

## Supplementary Material

Supplementary information

Crystal structure data
