# Supplementary material for: Between single ion magnets and macromolecules: a polymer/transition metal-based semi-solid solution
Source: Chem Sci. 2018 Aug 3;9(36):7277–86. doi: 10.1039/c8sc02277a (PMC6148685; doi:10.1039/c8sc02277a)
Supplement: Supplementary file 1 [file SC-009-C8SC02277A-s001.pdf]

Electronic Supplementary Material (ESI) for Chemical Science.
This journal is © The Royal Society of Chemistry 2018

Between Single Ion Magnets and macromolecules: polymer/transition metal–based semi–solid solution

Anna M. Majcher*[a], Paweł Dąbczyński[a], Mateusz M. Marzec[b], Magdalena Ceglarska[a], Jakub Rysz[a], Andrzej Bernasik[b,c], Shin-ichi Ohkoshi[d], Olaf Stefańczyk*[d]

[a] Faculty of Physics, Astronomy and Applied Computer Science, Jagiellonian University, Łojasiewicza 11, 30–348 Krakow, Poland

[b] Academic Centre for Materials and Nanotechnology, AGH University of Science and Technology, al. Mickiewicza 30, 30-049 Kraków, Poland

[c] Faculty of Physics and Applied Computer Science, AGH University of Science and Technology, al. Mickiewicza 30, 30-049 Kraków, Poland

[d] Department of Chemistry, School of Science, The University of Tokyo, 7-3-1 Hongo, Bunkyo-ku, Tokyo 113-0033, Japan

Electronic Supplementary Information

1. Structural data for the Co–based SIM compounds

Assemblies **1** and **2** crystallized in the monoclinic space group $P2_1/c$ and $C2/c$, respectively. Average Co–N and Co–Br distances for **1** equal 2.023 and 2.370 Å, respectively, while complex **2** shows very similar values of bond lengths equal 2.029 and 2.358 Å, respectively. Moreover, N–Co–N, average N–Co–Br, and Br–Co–Br angles for **1** adopt values of 108.4°, 107.4° and 118.4° in series. In case of the complex **2**, the N–Co–N and Br–Co–Br angles are smaller with values of 102.8° and 117.0°, respectively, while average N–Co–Br angle is slightly larger (109.0°). Observed structural parameters are consistent with values determined for other tetrahedral CoL_2Br_2 assemblies with N–donor monodentate aromatic ligands (Supplementary Table 1). Thermal ellipsoids diagram of asymmetric units of **1** and **2** are shown in Supplementary Figure 1 a) and b), respectively.

Supplementary Table 1. Comparison of selected structural parameters for **1**, **2** and other similar mononuclear assemblies.

Compound	REFCODE	Average distances (Å)		Average angles (°)		
		Co-N	Co-Br	N-Co-N	N-Co-Br	Br-Co-Br
**1**	-	2.023	2.370	108.4	107.4	118.4
**2**	-	2.029	2.358	102.8	109.0	117.0
$Co(L^1)_2Br_2$	BHMTZC	2.038	2.389	106.8	109.8	110.8
$Co(L^2)_2Br_2$	CINYAA	2.048	2.412	109.4	110.3	106.4
$Co(L^3)_2Br_2$	FOYWOG	2.002	2.384	98.1	113.4	105.7
$Co(L^4)_2Br_2$	LUKSUF	2.030	2.384	108.1	108.6	114.3
$Co(L^5)_2Br_2$	LULRAM	2.023	2.391	103.2	111.3	108.8
$Co(2,4\text{-dmpy})_2Br_2$	NADTOB	2.044	2.394	117.7	106.7	112.6
$Co(L^6)_2Br_2$	OQOKIP	2.003	2.392	112.1	107.5	114.7
$Co(L^7)_2Br_2$	OQOKOV	2.021	2.382	114.1	106.8	115.8
		2.022	2.381	104.2	109.3	115.3

$Co(2\text{-Brpy})_2Br_2$	POFYEO	2.050	2.386	108.4	110.7	105.7
$Co(L^8)_2Br_2$	REPFAV	2.022	2.431	108.0	112.0	100.8
$Co(qn)_2Br_2$	TUSQAB	2.045	2.389	112.7	107.7	113.4
	TUSQAB01	2.064	2.382	108.3	109.2	112.4
	TUSQAB02	2.047	2.381	111.9	107.9	113.3
$Co(3,5\text{-dmpyr})_2Br_2$	YADPAW	2.005	2.394	107.4	107.6	119.3
$Co(L^9)_2Br_2$	YARZEY	2.028	2.394	98.6	112.7	107.4
$Co(4,5\text{-dPhim})_2Br_2$	YASBEB	2.010	2.418	110.1	110.8	103.4
$Co(1,3\text{-btz})_2Br_2$	YOWZER	2.037	2.376	104.3	111.1	108.9
$Co(L^{10})_2Br_2$	MASXOW	2.044	2.442	110.8	110.8	102.2

REFCODE identifies the records from CSD V5.38 crystallographic data base.[1] 1,3–btz = 1,3–Benzothiazole, 2–Brpy = 2–bromopyridine, 2,4–dmpy = 2,4–dimethylpyridine, 3,5–dmpyr = 3,5–dimethyl–1H–pyrazole, 4,5–dPhim = 4,5–diphenyl–1H–imidazole, L^1 = 5–(2–hydroxyethyl)–4–methylthiazole, L^2 = N,N'–diethyl–6–phenoxy–1,3,5–triazine–2,4–diamine, L^3 = 4–acetyl–3–amino–5–methylpyrazole, L^4 = 1–hydroxyethyl–2–methyl–5–nitroimidazole, L^5 = 2–methyl–1H–benzimidazole, L^6 = 1–pentyl–1H–benzimidazole, L^7 = 1–propyl–1H–benzimidazole, L^8 = 3–hexyl–6–iminio–2–oxo–3,6–dihydropyrimidin–1(2H)–yl, L^9 = 1–methyl–4,5–diphenyl–1H–imidazole, L^{10} = 4–aminopyrimidin–2(1H)–one, qn = quinoline.

X–ray powder diffraction measurements showed that the powder diffraction patterns of polycrystalline samples of **1** and **2** are very similar (Supplementary Figure 1 c) and d)), which confirms the isostructural character of both compounds, and with simulated diffractograms for single crystals of **1** and **2**, which confirms the presence of the pure single phase. Slight differences in the intensities of diffraction peaks can be assigned to the effect of texturing.

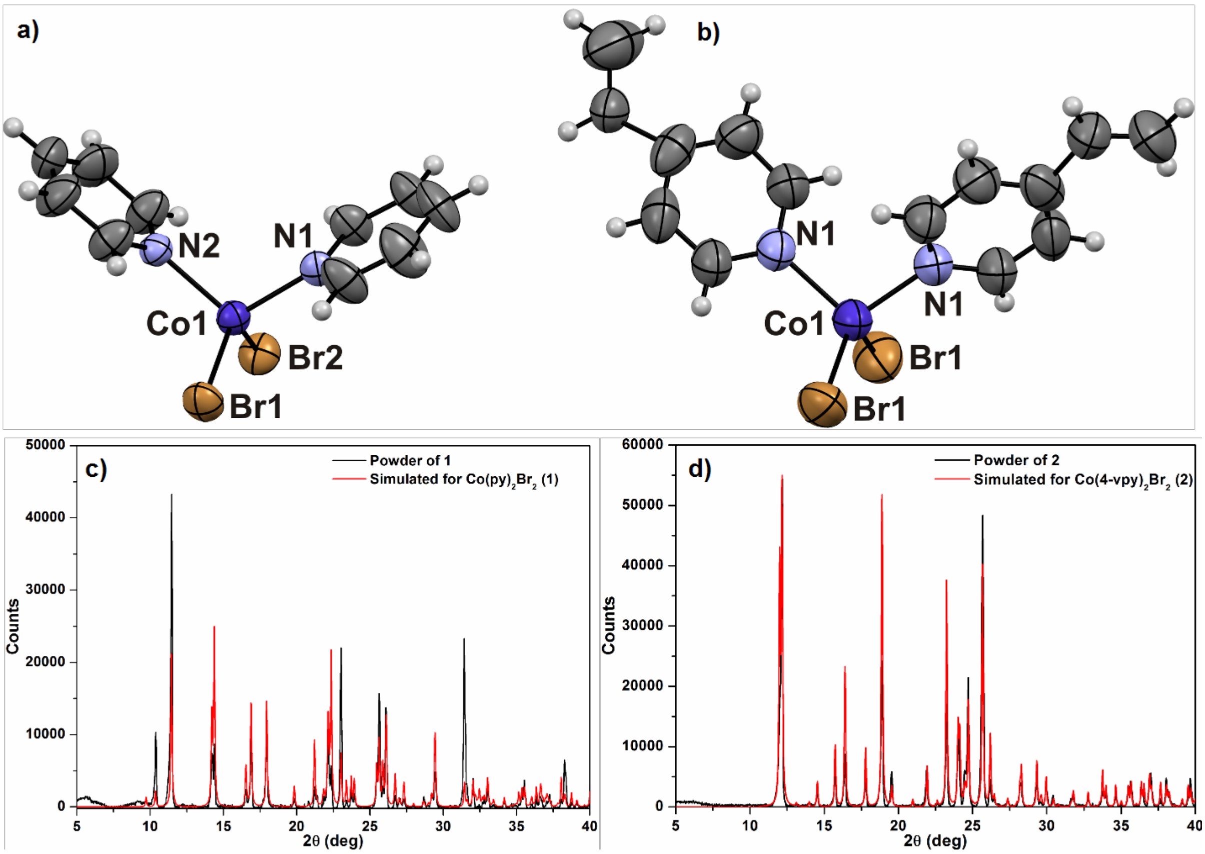

***Supplementary Figure 1**. Top: thermal ellipsoids diagram of the asymmetric unit of a) **1** and b) **2** with selected atoms labelling. Colours used: Br – brown, C – gray, Co – dark blue, N – blue. Thermal ellipsoids of 50% probability are shown. Bottom: comparison of room temperature X–ray powder diffraction data for **c)** powder of **1** (black) with simulation based on the single crystal structures of **1** (red) and **d)** powder of **2** (black) with simulation based on the single crystal structures of **2** (red).*

2. Photographs of the obtained mononuclear crystalline compounds.

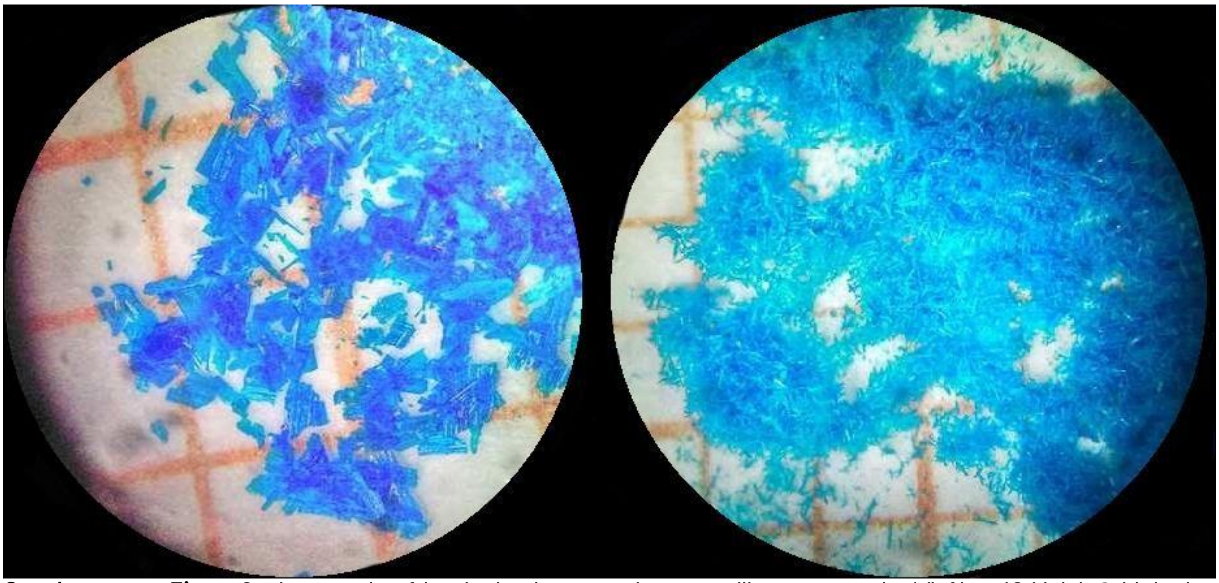

Supplementary Figure 2. *Photographs of the obtained mononuclear crystalline compounds: **1** (left) and **2** (right). Grid size is 1 mm.*

3. Comparison of microelemental analysis results and ICP+MS for 1, 2, example bulk P4VP with $CoBr_2$ and P4VP alone.

Supplementary Table 2. Microelemental analysis and ICP+MS results for **1**, **2**, and example bulk P4VP with $CoBr_2$ and P4VP alone. In the last two columns the number of P4VP structural units per one $CoBr_2$ obtained by these methods combined is compared with this value obtained from magnetic measurements to show perfect agreement.

Compound		Formula	M_W (g/mol)		Microelemental analysis				ICP+MS		P4VP str. units/$CoBr_2$	
					C (%)	H (%)	N (%)	Br (%)	Br (%)	Co (%)	EIM[b]	MGT[c]
**Co(py)$_2$Br$_2$ (1)**		$C_{10}H_{11}Br_2CoN_2O_{0.25}$ (**1** + 0.25 H_2O)	381.4	Calc.	31.49	2.77	7.34	41.90		15.45	–	–
				Found	31.63	2.87	7.38	41.78	41.67	15.51		
**Co(4vpy)$_2$Br$_2$ (2)**		$C_{12}H_{10}Br_2CoN_2O_2$ (**2**)	433.0	Calc.	33.29	2.33	6.47	36.91		13.61	–	–
				Found	33.24	2.37	6.42	36.83	36.75	13.48		
**P4VP str. units : $CoBr_2$ molar ratio in synthesis**	**2:1[a]**	$C_{33.25}H_{42}Br_2CoN_{4.75}O_{4.25}$ (**Co(P4VP)$_{4.75}$Br$_2$·4.25H$_2$O**)	795.0	Calc.	50.24	5.33	8.37	20.10		7.41	**4.8**	**4.7**
				Found	50.18	5.36	8.37	20.36	20.25	7.39		
	**5:1[a]**	$C_{45.5}H_{56.75}Br_2CoN_{6.5}O_{5.5}$ (**Co(P4VP)$_{6.5}$Br$_2$·5.5H$_2$O**)	1001.5	Calc.	54.57	5.71	9.09	15.96		5.88	**6.5**	**6.8**
				Found	54.42	5.78	9.10	16.15	15.91	5.76		
	**10:1[a]**	$C_{91}H_{118}Br_2CoN_{13}O_{13}$ (**Co(P4VP)$_{13}$Br$_2$·13H$_2$O**)	1820.7	Calc.	60.03	6.53	10.00	8.78		3.24	**13.0**	**12.9**
				Found	59.95	6.29	9.93	8.93	9.07	3.21		
	**20:1[a]**	$C_{119}H_{155}Br_2CoN_{17}O_{17}$ (**Co(P4VP)$_{17}$Br$_2$·17H$_2$O**)	2314.3	Calc.	61.76	6.75	10.29	6.91		2.55	**17.0**	**16.5**
				Found	61.76	6.51	10.22	7.03	6.96	2.44		
**P4VP**		$C_7H_{7.97}NO_{0.46}$ (**P4VP** + 0.46 H_2O)	113.5	Calc.	74.09	7.08	12.34	–		–	–	–
				Found	74.36	7.34	12.08	–	–	–		

[a] P4VP structural units: $CoBr_2$ molar ratio indicated in the name of the compound denotes the number of P4VP structural units per $CoBr_2$ units used in the synthesis.
[b] P4VP structural units: $CoBr_2$ molar ratio obtained from the collective results of elemental analysis and ICP+MS
[c] P4VP structural units: $CoBr_2$ molar ratio obtained from the value of magnetization at 1.8 K in 50 kOe compared with the same value for **1**.

4. Fourier–Transform Infrared spectroscopy (FTIR) measurements results for 1, 2, bulk P4VP with $CoBr_2$ and P4VP alone.

Supplementary Table 3. FTIR measurements results for **1**, **2**, bulk P4VP with $CoBr_2$ (measured for all the samples: x = 4.8, 6.5, 13.0, 17.0 with identical peak maxima positions in the spectra) and P4VP alone.

$Co(py)_2Br_2$ (1)	$Co(4vpy)_2Br_2$ (2)	$Co(P4VP)_xBr_2$	P4VP	Assignment
3407w(br), 3321m(br)		3370vs(br), 3230vs(br)	3375vs(br), 3244vs(br)	*ν*(O–H)
3108vw, 3088vw, 3064vw, 3043vw, 3026vw, 3002vw	3122vw, 3095vw, 3085vw, 3057vw, 3042vw, 3028vw, 3009vw, 2989vw	3053s(sh), 2925s, 2855m(sh)	3068w, 3048w, 3026w, 2989w, 2956m(sh), 2927m, 2899m(sh), 2854w	$\nu(C_{arom}$–H), *ν*(C–H)
	1949vw, 1891vw, 1848vw	1949vw	1943w	ν_{as}(C=C=C), *ν*(C=O)
1606vs	1616vs	1614vs, 1605vs	1599s	γ(O–H)
1488m, 1485m, 1449s(sh), 1446vs(sh), 1242w, 1218w(sh), 1215m	1544m, 1502m, 1428s, 1416s, 1301w, 1246w, 1223m, 1201m	1558m, 1501w, 1450w, 1422s, 1256vw, 1223w	1558m, 1496w, 1452w, 1419s, 1374vw, 1250vw, 1221m	$\delta(H–C_{arom}$–H), *ν*(C=C), *ν*(N–C), *ν*(C–C)
1160w, 1154w, 1067s, 1045s, 1014m, 1010w, 883vw, 759s, 750s, 696vs, 689vs, 643s, 442w, 425s, 418s	1065m, 1036w, 1021m, 991m, 987m(sh), 950m, 941m, 869w, 843s, 803w, 757w, 645w, 574w, 468w(sh), 458w	1068w, 1019w, 1007w, 830m, 759w, 745w, 625vw, 562w	1162vw, 1132vw, 1068m, 1000w(sh), 993w, 954vw, 820m, 759w, 745w, 558w	[$\gamma(C_{arom}$–H), *ν*(C–O), *γ*(C–H), *ν*(N–C)].

5. P4VP with $CoBr_2$ geometry optimization results.

Supplementary Table 4. Results of calculations for one P4VP chain coordinating one $CoBr_2$ unit with varying number of structural units between the units coordinating to the Co atom n = 0 – 6.

**Visualization of P4VP–$CoBr_2$ optimal geometry for n = 0 (top) to 6 (bottom)**	**Distances (Å)**		**Angles (°)**			**Results of geometry optimization**			
	**<Co–N>**	**<Co–Br>**	**N–Co–N**	**Br–Co–Br**	**<N–Co–Br>**	**Energy (au)**	**ΔE (au)**	**Grad Norm**	**‖Max Grad(i)‖**
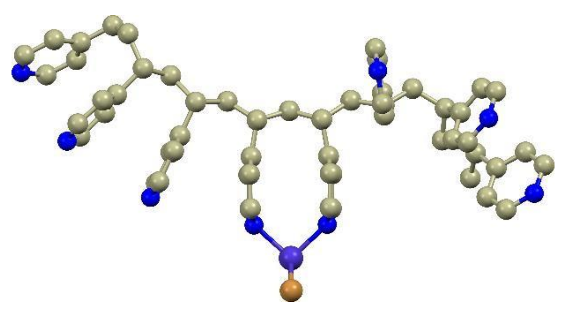	2.019	2.367	95.38	112.06	112.17	0.268695	$-1.9590\cdot10^{-7}$	0.000260	$7.1\cdot10^{-5}$
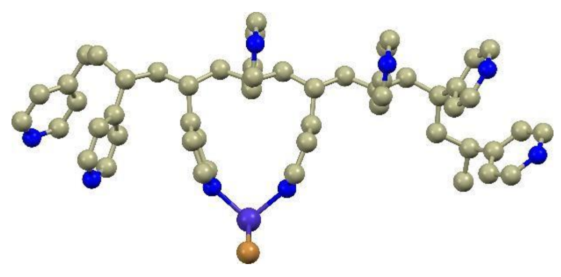	2.020	2.367	98.82	111.56	111.21	0.231294	$-9.8942\cdot10^{-7}$	0.000240	$6.9\cdot10^{-5}$
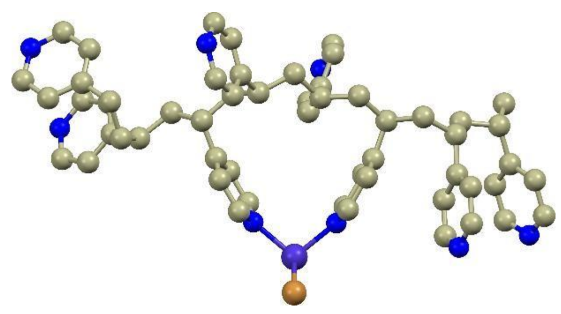	2.020	2.368	101.00	111.18	110.78	0.218175	$-2.5508\cdot10^{-7}$	0.000291	$8.4\cdot10^{-5}$
	**Distances (Å)**		**Angles (°)**			**Results of geometry**			
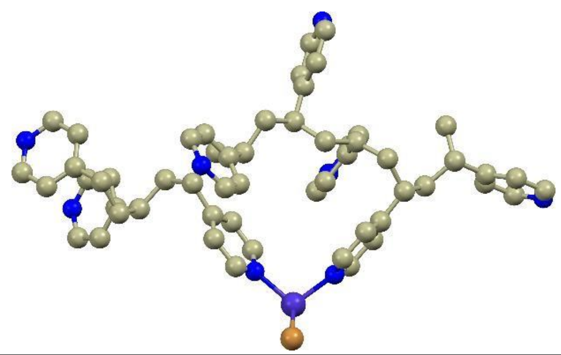	2.021	2.367	103.27	110.80	110.20	0.229744	$-1.7199\cdot10^{-7}$	0.000314	$8.2\cdot10^{-5}$

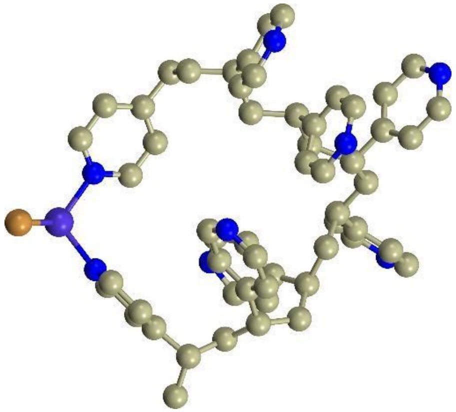	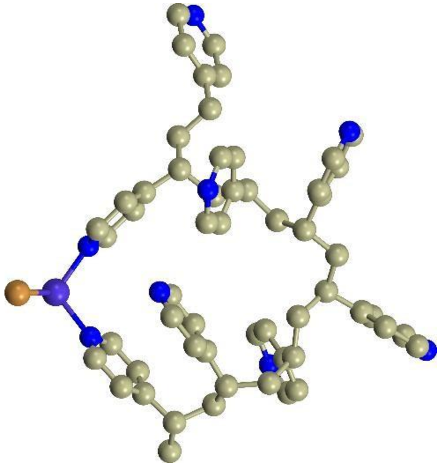	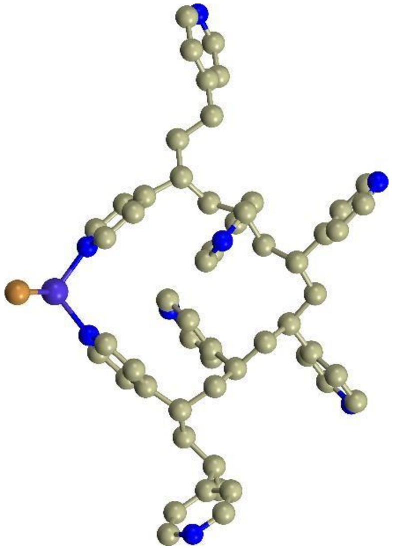
2.025	2.029	2.029
2.367	2.367	2.368
107.13	107.02	106.19
110.02	110.12	110.28
109.61	109.32	109.50
0.217770	0.218926	0.209615
$-1.9742 \cdot 10^{-7}$	$-2.4670 \cdot 10^{-8}$	$-2.6739 \cdot 10^{-7}$
0.000254	0.000372	0.000387
$7.4 \cdot 10^{-5}$	$8.3 \cdot 10^{-5}$	$7.5 \cdot 10^{-5}$

6. AC magnetic susceptibility *vs*. frequency for 1 and 2.

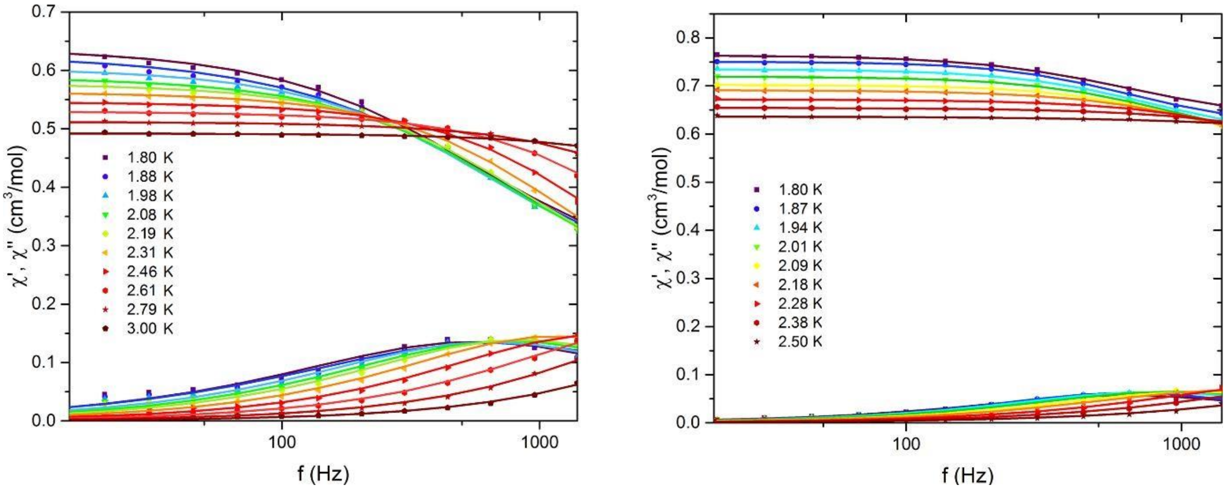

Supplementary Figure 3. *AC magnetic susceptibility in H_{DC} = 2.5 kOe for **1** (left) and **2** (right) versus frequency. H_{AC} = 3 Oe. Solid lines represent respective Cole–Cole model fits performed simultaneously for χ' and χ''.*

7. AC magnetization versus frequency for the bulk $CoBr_2$–P4VP.

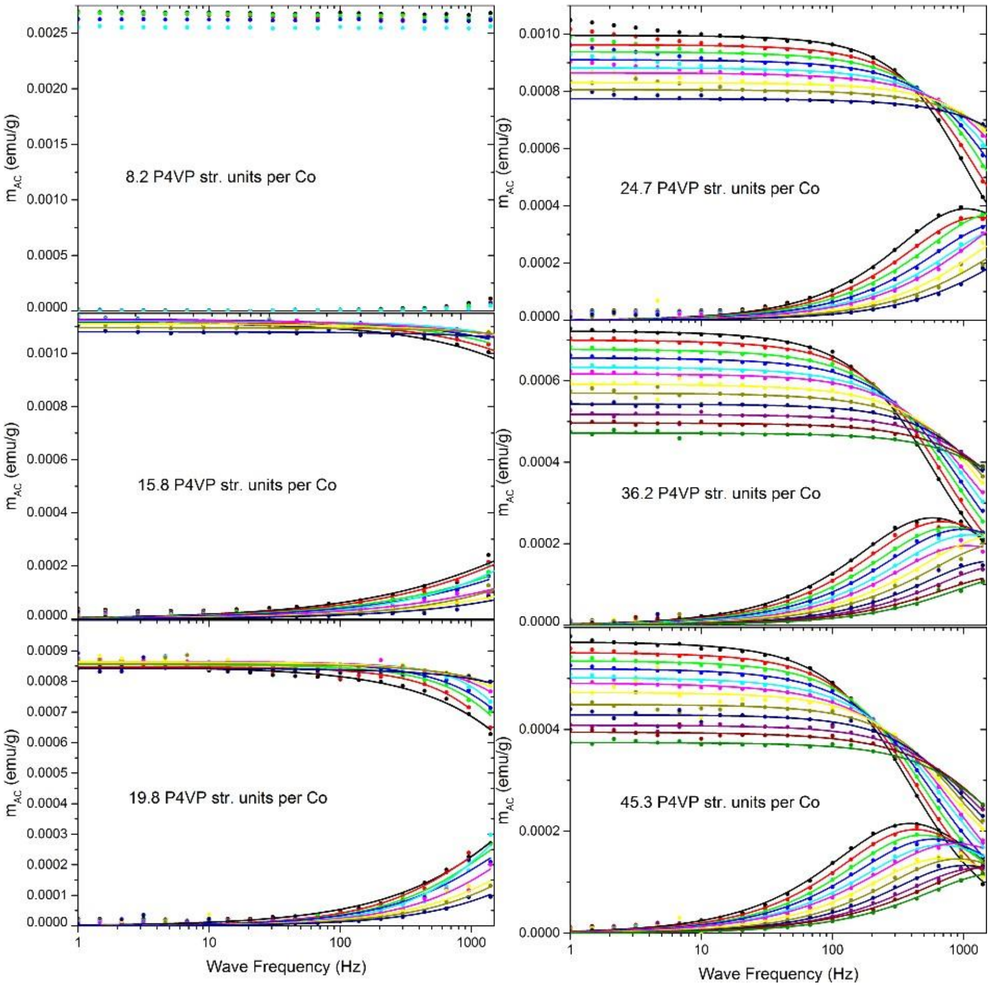

Supplementary Figure 4. *AC magnetization in H_{DC} = 2.5 kOe as a function of frequency for bulk P4VP with $CoBr_2$ in varying molar ratios. H_{AC} = 3 Oe. Solid lines represent respective Cole–Cole model fits performed simultaneously for m' and m''.*

8. Thin film roughness from AFM measurements. The roughness of the obtained topographies was analysed using the WSxM 4.0 software.[2] The results are gathered in Supplementary Table 5.

Supplementary Table 5. Comparison of the RMS roughness for the obtained thin films.

Surface	RMS roughness
As cast	1.15 nm
Treated with acetonitrile	0.97 nm
Treated with $CoBr_2$ acetonitrile solution	12.62 nm

9. XPS analysis for unmodified and modified P4VP thin films and the bulk P4VP with $CoBr_2$.

Surface concentrations of C, N, Co and Br (atomic concentration in %) obtained from XPS measurements for all systems studied are listed in Supplementary Table 6. The untreated, as cast sample shows slightly higher carbon concentration (90.6 %) than theoretically expected (87.5%) but this can be attributed to the adventitious carbon usually found on the surface of most air exposed samples. The C 1s and N 1s spectra for as cast P4VP thin film are presented in Supplementary Figure 5. The C 1s spectrum can be fitted with two components arising from aliphatic carbon and carbon atoms in pyridine (both at 284.8 eV) and C–N bonds in pyridine units (285.6 eV). The N 1s spectrum consists of one major, symmetrical peak at 399.0 eV which can be ascribed to nitrogen in pyridine ring.[3] The other two minor components at 400 eV and 401.5 eV can be attributed to protonated pyridine units[4] and possibly N–oxide of pyridine or another form of adsorbed nitrogen on the surface,[5] respectively. However, their contribution in overall N 1s spectrum is low and amounts to ca. 3.0 % each. The spectra for P4VP film treated with acetonitrile (the second row in Supplementary Figure 5) is similar to that obtained for the as cast one with just a slight increase in nitrogen concentration (11.4%) now being very close to expected one (12.5%). Concerning the spectra shapes and lines positions it might be concluded that treatment with acetonitrile did not change polymer composition. Doping with $CoBr_2$ (the third row in Supplementary Figure 5) produced a new, highly energetic N 1s peak at binding energy about 400.1 eV which was previously detected for P4VP doped with either Pd[6] or Pt[7] complexes, and was ascribed to N atoms coordinated with these metals. Here peaks at BE > 400 eV are dominant and represent approximately 60.1% of the overall N 1s spectra for $CoBr_2$ doped P4VP film, respectively and can be ascribed to Co atoms coordinating nitrogen in pyridine units. The Co 2p spectra (Supplementary Figure 6) for $CoBr_2$ treated polymer film show same envelope with main line situated at 780.2 eV which indicate Co^{2+} state of the metal.[8] The Br 3d spectrum for $CoBr_2$–treated P4VP shows one single doublet structure with main peak centered at 68.0 eV originating from the presence of metal bromide.[9] What needs to be underlined is that the spectra measured for bulk P4VP with $CoBr_2$ (Supplementary Figure 7) are consistent with the ones obtained for thin layers of P4VP doped with $CoBr_2$.

Supplementary Table 6. Surface composition (atomic concentration in %) determined by XPS.

Sample	C	N	Co	Br	N/Co
as_cast	90,6	9,4	–	–	–
acetonitrile	88,6	11,4	–	–	–
$CoBr_2$	84,3	9,0	4,4	2,3	2,05
P4VP theor.	***87,5***	***12,5***	–	–	–

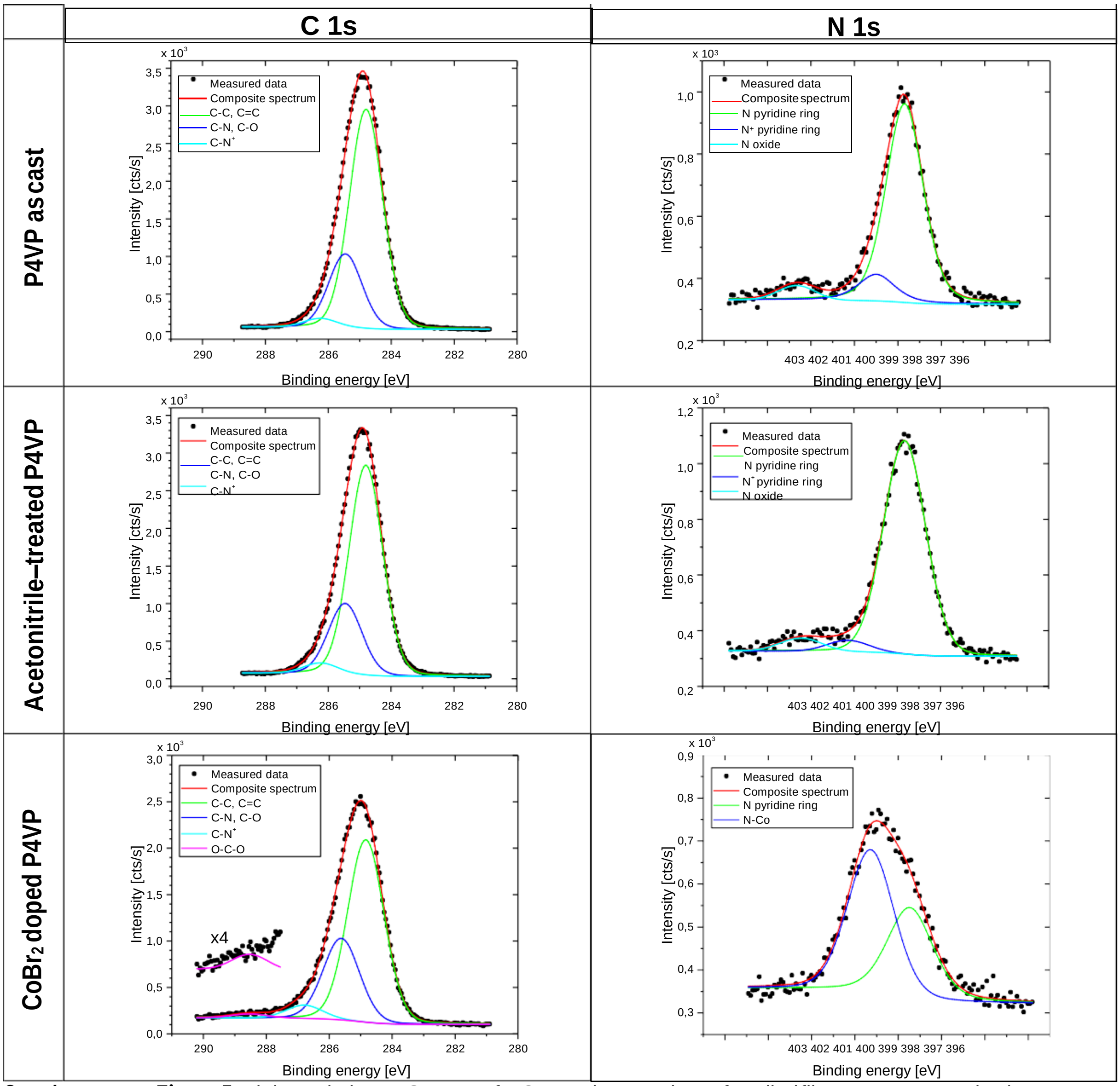

Supplementary Figure 5. *High resolution XPS spectra for C 1s and N 1s regions of studied films: P4VP as cast (top), acetonitrile–treated (middle) and treated with acetonitrile solution of $CoBr_2$ (bottom).*

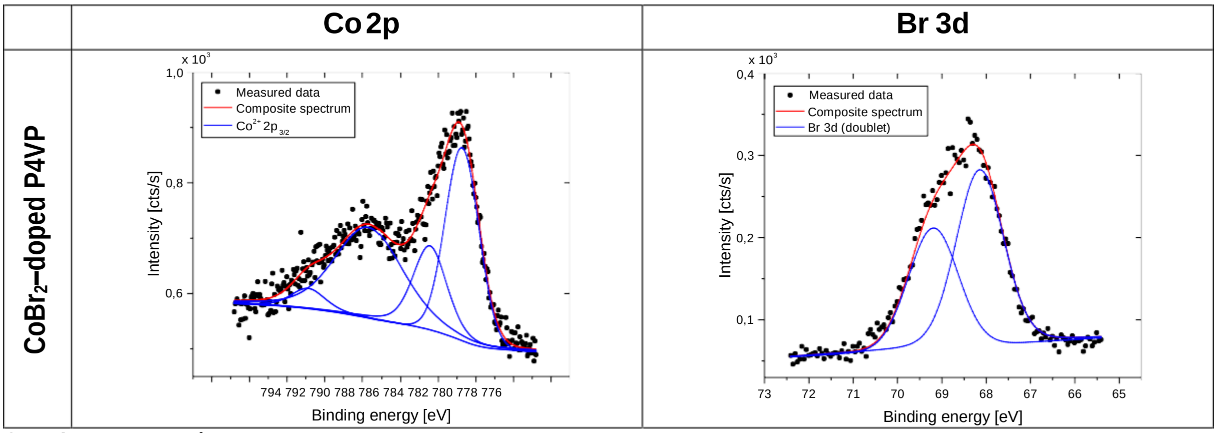

Supplementary Figure 6. *High resolution XPS spectra of Co 2p and Br 3d regions for the $CoBr_2$–doped P4VP thin films.*

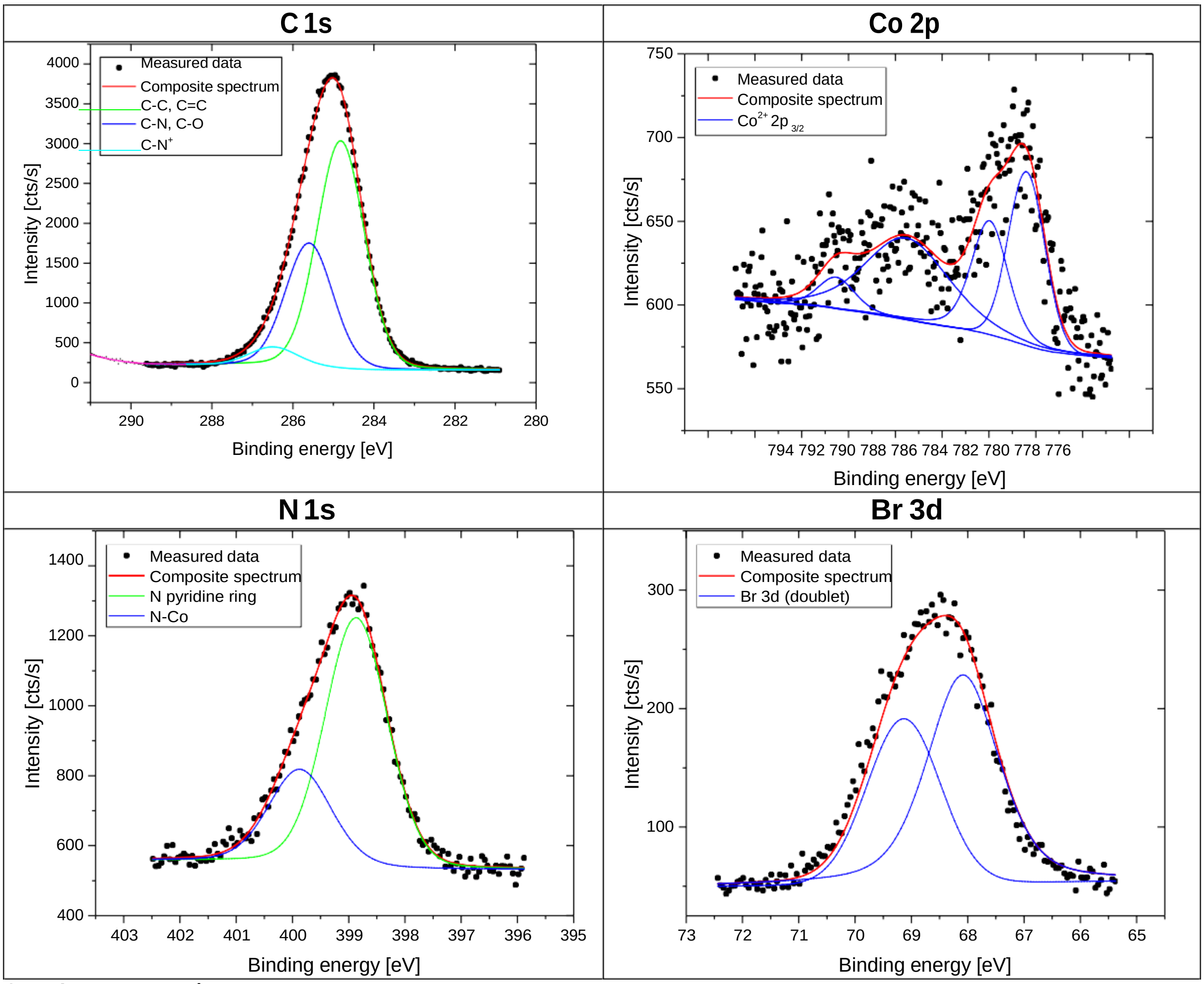

Supplementary Figure 7*. High resolution XPS spectra for bulk P4VP with $CoBr_2$ in 10:1 molar ratio.*

10. Ellipsometry measured film thicknesses.

Supplementary Table 7. Film thicknesses obtained from ellipsometry measurements for films: as cast, acetonitrile–treated and treated with $CoBr_2$ acetonitrile solution.

Sample	Film Thickness [nm]	Film Thickness Error [nm]
P4VP as cast	180.5	± 13.5
P4VP acetonitrile–treated	181.2	±14.2
P4VP $CoBr_2$ acetonitrile solution–treated	189.3	±13.8

11. SIMS supplementary data for the obtained films.

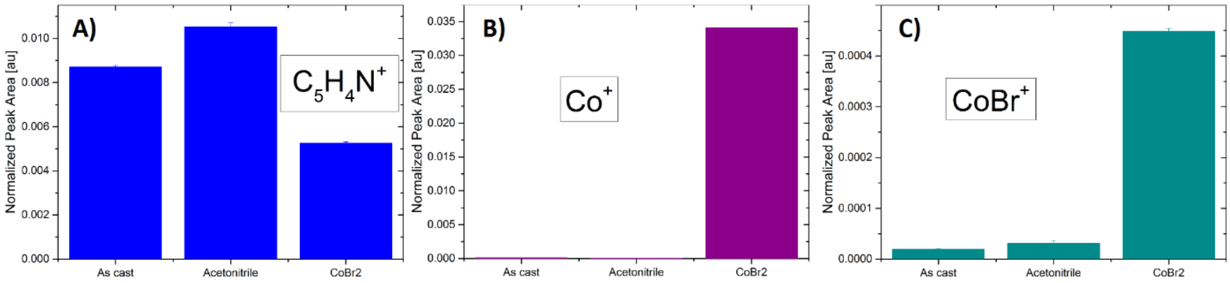

***Supplementary Figure 8**. Normalized area of selected peaks from positive mass spectra for layers as cast, treated with acetonitrile only, and treated with a solution of $CoBr_2$ in acetonitrile. Data presented for ions: A) $C_5H_4N^+$, B) Co^+, C) $CoBr^+$.*

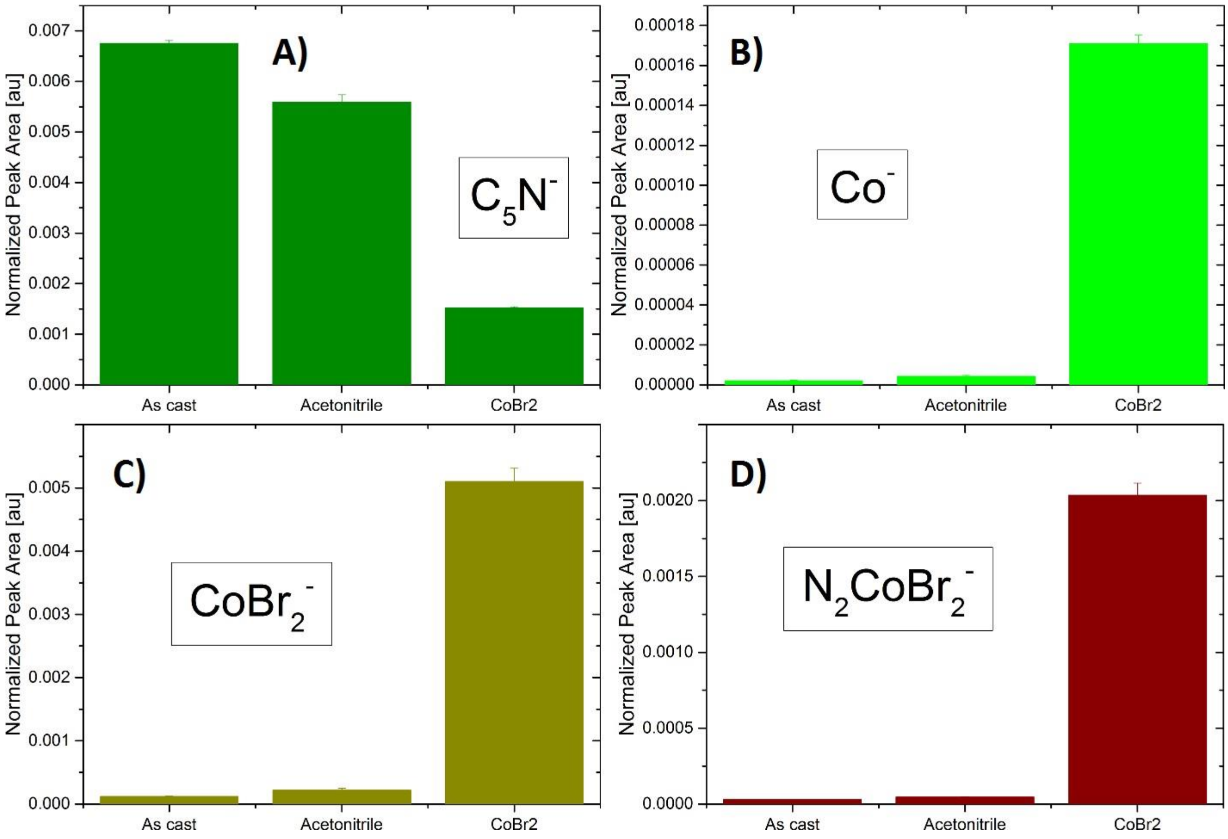

***Supplementary Figure 9**. Normalized area of selected peaks from negative mass spectra for layers as cast, treated with acetonitrile only, and treated with a solution of $CoBr_2$ in acetonitrile. Data presented for ions: A) C_5N^-, B) Co^-, C) $CoBr_2^-$, D) $N_2CoBr_2^-$.*

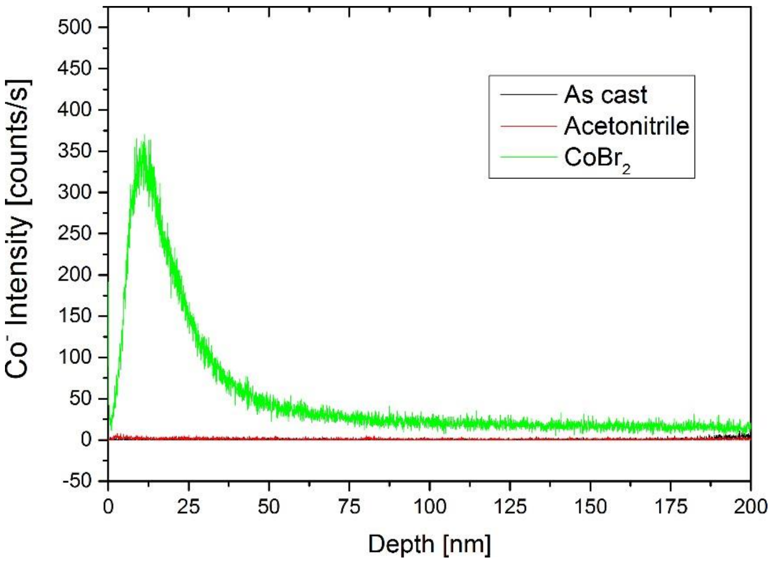

Supplementary Figure 10. *Depth profile of Co^- from sample as cast (black line), acetonitrile-treated layer (red line), and layer treated with $CoBr_2$ solution in acetonitrile (green line).*

Depth profiles calibration were obtained based on film thickness measured with the ellipsometry technique. Time needed to completely sputter the sample were determined by the Si^- signal. The total thin film thickness and the total sputtering time allows us to determine the sputtering rate for all samples. Time scale was converted to depth scale assuming constant sputtering rate.

1 C. R. Groom, I. J. Bruno, M. P. Lightfoot, S. C. Ward, The Cambridge Structural Database. *Acta Cryst*. 2016, **B72,** 171.
2 I. Horcas, R. Fernández, J. M. Gómez–Rodríguez, J. Colchero, J. Gómez–Herrero, A. M. Baro, *Rev. Sci. Instrum.* 2007, **78**, 013705–1.
3 G. Beamson, D. Briggs, *J. Chem. Educ.* 1993, **70**, A25.
4 N. S. Gill, R. H. Nuttall, D. E. Scaife, D. W. A. Sharp, *J. Inorg. Nucl. Chem.* 1961, **18**, 79.
5 L. Wachowski, J. W. Sobczak, M. Hofman, *Appl. Surf. Sci.* 2007, **253**, 4456.
6 J. P. Mathew, M. Srinivasan, *Eur. Polym. J.* 1995, **31**, 835.
7 A. Drelinkiewicz, J. W. Sobczak, E. Sobczak, M. Krawczyk, A. Zięba, A. Waksmundzka-Góra, *Mater. Chem. Phys.* 2009, **114**, 763.
8 M. C. Biesinger, B. P. Payne, A. P. Grosvenor, L. W. M. Lau, A. R. Gerson, R. St. C. Smart, *Appl. Surf. Sci.* 2011, **257**, 2717.
9 W. E. Morgan, J. R. Van Wazer, W. J. Stec, *J. Am. Chem. Soc.* 1973, **95**, 751.
